# Supplementary material for: Automated Generation of Synoptic Reports from Narrative Pathology Reports in University Malaya Medical Centre Using Natural Language Processing
Source: Diagnostics (Basel). 2022 Apr 1;12(4):879. doi: 10.3390/diagnostics12040879 (PMC9027647; doi:10.3390/diagnostics12040879)
Supplement: Supplementary file 1 [file diagnostics-12-00879-s001.zip › Supplementary Files/Pathology checklist-style reporting template/PathologyReporting.php]

Pathology Reporting


php date\_default\_timezone\_set('Asia/Kuala\_Lumpur'); $today = date("Y-m-d"); ?

View Reports
Pathology Reporting

## --- Pathology Reporting ---

**Patient RN :**

**Report ID :**

**Date :**

  

|  |
| --- |
| **Specimen 1 :**    ---  **Interpretation** |
| Type of Procedure  : Yes      No |
| Needle biopsy    Excision    Total mastectomy    Other (Specify) |
| Laterality  : Yes      No |
| Left      Right |
| Histologic Type(s)  : Yes      No |
| No residual invasive carcinoma    Invasive carcinoma of no special type    Micro-invasive carcinoma    Invasive lobular carcinoma    Invasive carcinoma with mixed ductal and lobular features    Tubular carcinoma    Invasive cribriform carcinoma    Mucinous carcinoma    Invasive micropapillary carcinoma    Apocrine adenocarcinoma    Metaplastic carcinoma    Encapsulated papillary carcinoma with invasion    Solid papillary carcinoma with invasion    Intraductal papillary adenocarcinoma with invasion    Adenoid cystic carcinoma    Neuroendocrine tumor    Neuroendocrine carcinoma    Invasive carcinoma, type cannot be determined    Invasive carcinoma with features of (specify)    Other histologic type (specify) |
| Histologic Grade (Bloom and Richardson's)  : Yes      No |
| Grade  : Select one 1  2 3    Grade cannot be determined (explain) |
| **Macroscopy** |
| Tumor(s)  : Yes      No |
| **Tumor 1**    Type  =    Size  =    + |
| Margin(s)  : Yes      No |
| **Margin 1**    Type  =    Anterior  =  Deep  =  Superior  =  Inferior  =  Medial  =  Lateral  =  Posterior  =  Superficial  =  Peripheral  =  Axis  =  Other (specify margin)  =    + |
| Skin Involvement  : Yes      No |
| **Microscopy** |
| DCIS Nuclear Grade  : Yes      No |
| Grade  : Select one Low  Intermediate High    Other (Specify)  : |
| DCIS Pattern(s)  : Yes      No |
| Cribriform    Micropapillary    Papillary    Solid    Flat or clinging    Comedo    Other DCIS pattern (specify) |
| Lymphovascular Invasion  : Yes      No |
| Paget's Disease  : Yes      No |
| Skin Involment  : Yes      No |
| Regional Lymph Node(s)  : Yes      No |
| Total number of lymph nodes examined  :  Number of lymph nodes involved by tumour  : |
| Tumor(s)  : Yes      No |
| **Tumor 1**    Type  =    Size  =    + |
| Margin(s)  : Yes      No |
| **Margin 1**    Type  =    Anterior  =  Deep  =  Superior  =  Inferior  =  Medial  =  Lateral  =  Posterior  =  Superficial  =  Peripheral  =  Axis  =  Other (specify margin)  =    + |
| Immunohistochemistry  : Yes      No |
| Estrogen Receptor  : Positive      Negative      Other (Specify)  Progesterone Receptor  : Positive      Negative      Other (Specify)  HER2  : Positive      Negative      Equivocal       Score |

 Add More |

php
date\_default\_timezone\_set('Asia/Kuala\_Lumpur');
$date = date('Y/m/d H:i:s');
$report = strval(preg\_replace( '/[\W]/', '', $date));
?
