# Supplementary material for: Automated Generation of Synoptic Reports from Narrative Pathology Reports in University Malaya Medical Centre Using Natural Language Processing
Source: Diagnostics (Basel). 2022 Apr 1;12(4):879. doi: 10.3390/diagnostics12040879 (PMC9027647; doi:10.3390/diagnostics12040879)
Supplement: Supplementary file 1 [file diagnostics-12-00879-s001.zip › Supplementary Files/Pathology checklist-style reporting template/PathologyReporting_ViewDatabase.php]

BIRADS Standard Reporting

View Reports
Pathology Reporting  
  
Filter:

Filter by:   
Breast Composition- A
- B
- C
- D
- Not fill
  
Indication- Screening
- Diagnostic
- Follow up
- Not fill
  
Type
Mammogram- Mass
- Asymmetry
- Architectural distortion
- Benign calcification
- Suspicious calcification
- Associated features
  
Ultrasound- Mass
- Calcifications
- Associated features
- Axillary lymph node
  
Sort by- Report No
- RN
- Breast composition

## Breast Imaging Reporting and Data System (BIRADS)

Delete Database

php
$servername = "localhost";
$username = "root";
$password = "";
@$dbname = 'pathology\_extracted';
@$conn = mysqli\_connect($servername, $username, $password, $dbname);
if (!$conn) {
die("Connection failed: No database exists. Please try to insert new record. ");
}
$result = mysqli\_query($conn,"SELECT \* FROM report\_info");
echo "<table border='0'| No. | Patient RN | Report ID | Examination Date |  |
| --- | --- | --- | --- | --- |
";
while($row = mysqli\_fetch\_array($result))
{
$id=$row["report\_no"];
echo " " . $row["No"] . " |";
echo " " . $row["patient\_rn"] . " |";
echo " " . $row["report\_no"] . " |";
echo " " . $row["date"] . " |";
echo " X |";
echo "";
}
echo "";
mysqli\_close($conn);
?>
