# Supplementary figures and images for: Automated Generation of Synoptic Reports from Narrative Pathology Reports in University Malaya Medical Centre Using Natural Language Processing
Source: Diagnostics (Basel). 2022 Apr 1;12(4):879. doi: 10.3390/diagnostics12040879 (PMC9027647; doi:10.3390/diagnostics12040879)

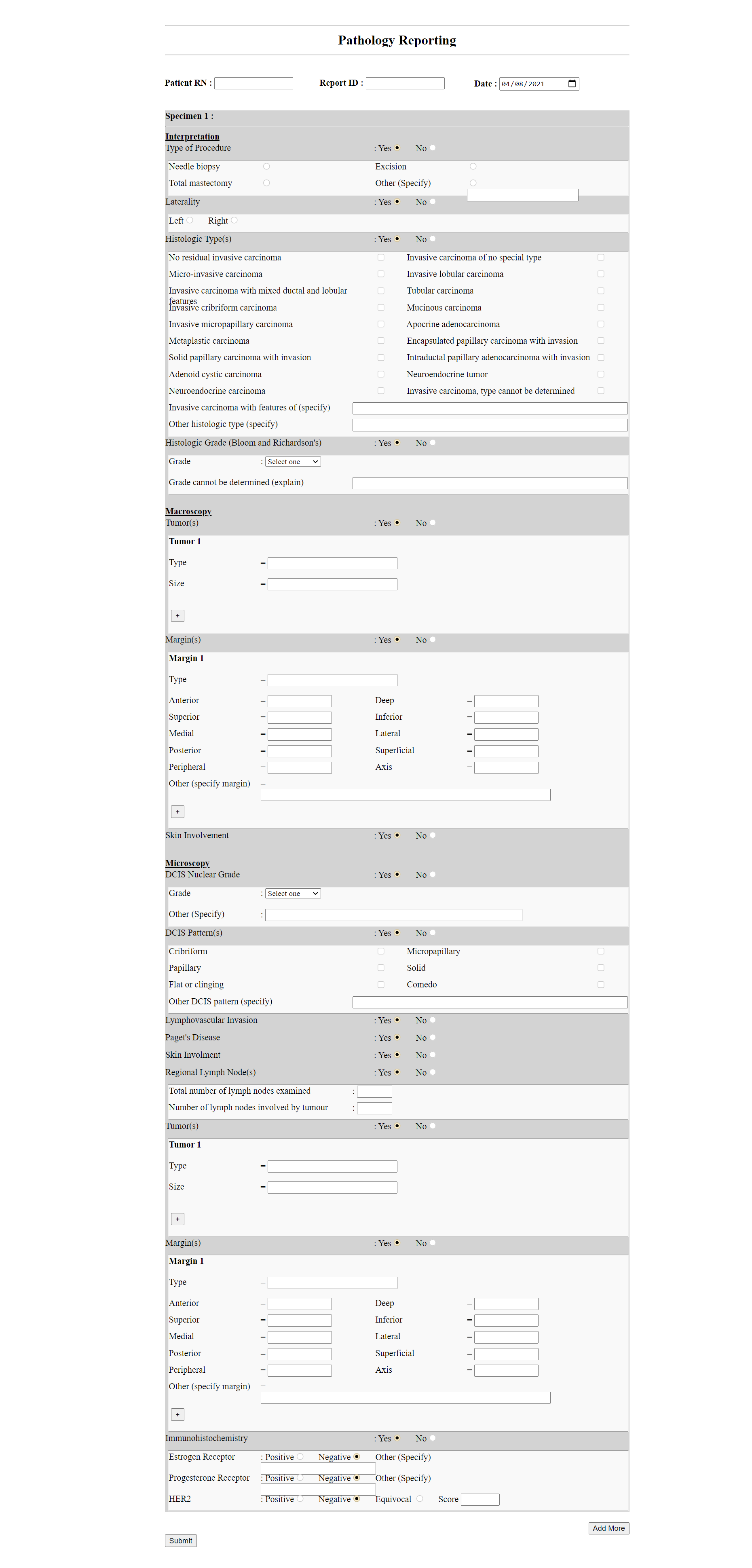

Supplement: Supplementary file 1 [file diagnostics-12-00879-s001.zip › Supplementary Files/Pathology checklist-style reporting template/screencapture of Pathology Reporting Template.png]
